# Migration of fully deployed stent after intracoronary glyceryl trinitrate administration: An unusual percutaneous coronary intervention complication

**DOI:** 10.12669/pjms.293.3205

**Published:** 2013

**Authors:** Murat Celik, Uygar Cagdas Yuksel, Yalcin Gokoglan

**Affiliations:** 1 Murat Celik, MD, Department of Cardiology, Gulhane Military Medical Faculty, Ankara-Turkey.; 2 Uygar Cagdas Yuksel, MD, Department of Cardiology, Gulhane Military Medical Faculty, Ankara-Turkey.; 3 Yalcin Gokoglan, MD, Department of Cardiology, Gulhane Military Medical Faculty, Ankara-Turkey.

**Keywords:** Right coronary artery, Intracoronary glyceryl trinitrate, Migration of deployed stent

## Abstract

Stent embolization is a rare complication of coronary stenting. If left untreated, it may lead to devastating consequences. Although there is much known about stent embolization, data about migration of stent after deployment is limited. We report an unusual case of a deployed stent migration into the distal part of right coronary artery after intracoronary glyceryl trinitrate administration during percutaneous coronary intervention.

## INTRODUCTION

Although coronary stenting has become an established and effective modality, some undesirable events such as stent embolization may still occur. Although it is a very rare event, stent embolization may lead to devastating consequences such as acute closure of the affected vessel, coronary thrombosis, and myocardial infarction.^1^ There are many case reports of stent embolization. Nevertheless, the number of case reports describing the migration of stent after deployment are limited in literature. We report an unusual case of migration of deployed stent into the distal part of right coronary after intracoronary glyceryl trinitrate administration.

## CASE REPORT

A 61-year-old man was admitted to our cardiology unit for the evaluation of his chest pain which occurred one week previously. His treadmill exercise test was positive and a coronary angiography (CAG) was performed. CAG revealed a significant stenotic lesion (70%) at the mid segment of right coronary artery (RCA). Percutaneous coronary intervention (PCI) was undertaken for the significant lesion. The RCA was engaged with a 6F JR 3.5 guiding catheter. The lesion was crossed with a 0.014” floppy wire and a 2.75x14 mm bare metal stent was successfully deployed at a pressure of 8 atm (nominal pressure). After stent deployment, coronary vasospasm of the vessel segment adjacent to proximal tip of the stent was observed. Then, we decided to administer intracoronary glyceryl trinitrate at a recommended dose (100 μg) in order to resolve this coronary vasospasm. After glyceryl trinitrate administration we noticed that coronary vasospasm was resolved and interestingly, the stent deployed at mid-RCA was not on-site. We examined carefully and noticed that the stent had migrated forward and embolized distally, being lodged just before posterior descending artery branch. The patient was asymptomatic, and either acute closure of the affected vessel or dissection was not present. Also, the residual lesion at the site where we deployed the stent initially was not significantly stenotic anymore. So, we did not decide any intervention or surgery. The migrated stent was inflated with a smaller size of balloon (2.5x12 mm balloon) through the stent. The procedural result was excellent with TIMI-3 flow distally. He was discharged in very good condition with an optimal medical treatment.

## DISCUSSION

Coronary stent embolization is an uncommon but potentially hazardous complication of PCI. The incidence ranges from 0.32% to 8.3%.^2^ Stent embolizations are observed especially in angulated, calcified and tortuous lesions. Also, physicians’ experience is important. Although many case reports are published about embolisation of stent before deployment^3^, the reports of migration of stent after deployment are rare. 

**Fig.1:**
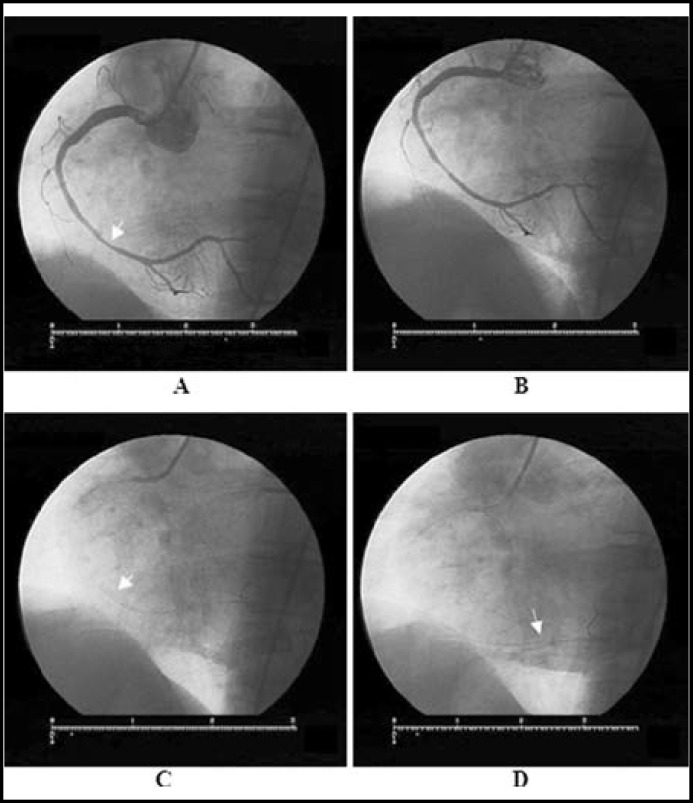
All selective right coronary angiograms in left anterior oqlique (LAO) view

**A: **A signficant stenotic lesion at the middle segment of right coronary artery (yellow arrow),

**B:** After implantation of a 14-mm-long 2.75 mm bare metal stent coronary vasospasm of the vessel segment adjacent to proximal end of the stent is seen,

**C: **The sturts of deployed stent is seen at mid-RCA before acute marginal branch (yellow arrow),

**D: **Then the struts of deployed stent is seen just before posterior descending artery branch after intracoronary glyceryl trinitrate administration (yellow arrow).

**Fig.2:**
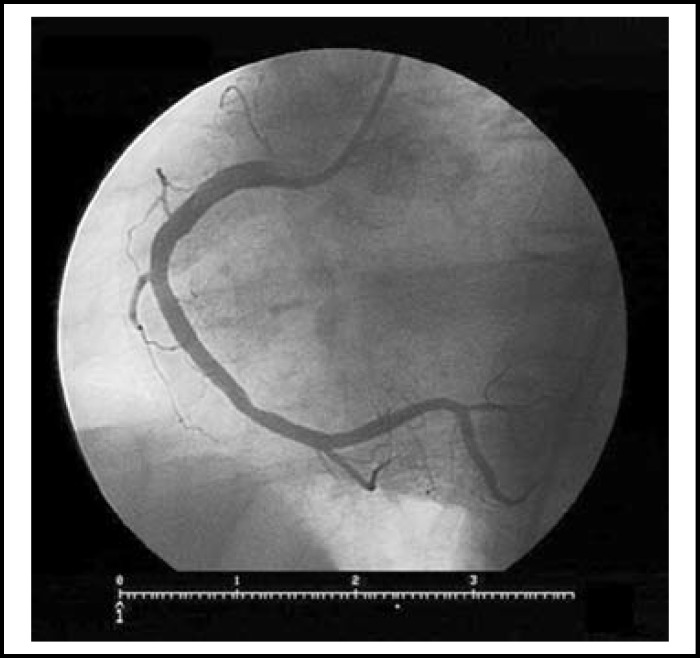
After inflation of migrated stent with a smaller size balloon at its lodged site, the procedural result was excellent with TIMI-3 flow distally. Also, the residue lesion at the site of firstly stented segment of RCA was no longer significantly stenotic

Pathophysiology of coronary vasoconstruction during PCI is explained by several mechanisms.^4^ The immediate effect of stenting on microvascular endothelial function is one of them. There is a down-regulation of eNOS, and increased release of the vasoconstrictors serotonin and endothelin-1 in a vessel exposed to low shear stress.^4^^-^^7^ Glyceryl trinitrate acts by releasing nitric oxide and known as a potent vasodilator. The use of intacoronary glyceryl trinitrate is beneficial in resolving coronary vasospasm. Also, intracoronary glyceryl trinitrate has been widely used to measure the reference vessel diameter before stenting. However, in the current era of PCI, this procedure has not been used routinely before every PCI.

There is no published data about migration of deployed stent after administration of intracoronary glyceryl trinitrate. Hence, in view of showing the migration of deployed stent after administration of intracoronary glyceryl trinitrate, our case report is the first in literature. Although the exact mechanism of migration was not known clearly, we suspect that the main reason for migration of deployed stent was under measurement of vessel diameter and so the improper embedding of stent struts at all into the arterial wall Subsequently, when we administered intracoronary glyceryl trinitrate, the stent had migrated distally. If we had administered intracoronary glyceryl trinitrate for measuring vessel diameter before stenting, the stent might not have migrated distally after deployment.

Treatment of stent embolisation may vary. Effort should be made to retrieve the lost stent properly. If it is not possible, deployment of the embolized stent with a smaller size of balloon or crushing technique may be an alternative to retrieval.^2^ Cardiac bypass surgery should be considered when the retrieval or deployment method fails. Either acute closure of the affected vessel or coronary thrombosis was not present in our patient. Thus, we did not consider retrieval of migrated stent or surgery. We left it at the migrated site after inflation with a smaller size of balloon.

In conclusion, to the best of our knowledge this case report showing migration of deployed stent after intracoronary glyceryl trinitrate administration is the first in literature. Thus, interventional cardiologists should not give up the possibility of migration of stent deployed successfully during PCIs. Also this case report emphasizes the measurement of vessel diameter by administration of intracoronary glyceryl trinitrate before stenting, especially in RCA.
